# Factors associated with the intention to practise family planning among antenatal women with risk of gestational diabetes mellitus in Klang Health District: A cross-sectional study

**DOI:** 10.51866/oa.487

**Published:** 2024-06-28

**Authors:** Baharuddin Siti Athirah, Sumsusdin Mohd Hafiz, Ley Na Dong, Pei Sean Lim, Shahul Hameed Nasrinsa, Md Nawawi Nor Aliah, A'ffan Nur Akmal, Kanapathy Shanmuga Kritika, Mohamad Fadzilah, Ai Theng Cheong

**Affiliations:** 1 MBBS, MMed, PhD, Department of Family Medicine, Faculty of Medicine and Health Sciences, Universiti Putra Malaysia, Serdang, Selangor, Malalysia. Email: cheaitheng@upm.edu.my; 2 MBBS, icFRACGP, Klinik Kesihatan Kapar, Ministry of Health Malaysia, Selangor, Malaysia. Email: athirah137@yahoo.com; 3 MD, icFRAGCP, Klinik Kesihatan Pandamaran, Jalan Raja Muda Musa, Kawasan 10, Pelabuhan Klang, Selangor, Malaysia.; 4 MBBS, icFRAGCP, Klinik Kesihatan Pandamaran, Jalan Raja Muda Musa, Kawasan 10, Pelabuhan Klang, Selangor, Malaysia.; 5 MD, icFRAGCP, Klinik Kesihatan Anika Klang, 112 Jalan Pegawai Off Jalan Tengku, Kelana, Klang, Selangor, Malaysia.; 6 MD, icFRAGCP, Klinik Kesihatan Pulau Indah, JKR 1353 Jalan Rahmat Off Persiaran, Masjid Sultan, Pulau Indah, Selangor, Malaysia.; 7 MD, icFRAGCP, Klinik Kesihatan Pandamaran, Ministry of Health Malaysia, Jalan Raja Muda Musa, Kawasan 10, Pelabuhan Klang, Selangor, Malaysia.; 8 MD, icFRAGCP, Klinik Kesihatan Anika Klang, 112 Jalan Pegawai, Off, Jalan, Tengku Kelana, Klang, Selangor, Malaysia.; 9 MBBS, icFRAGCP, Klinik Kesihatan Bukit Kuda, Jalan Batu Tiga Lama, Kawasan 15, Klang, Selangor, Malaysia.; 10 MBBCh BAO, MMed, Department of Family Medicine, Faculty of Medicine and Health Sciences, Universiti Putra Malaysia, Serdang, Selangor, Malalysia.

**Keywords:** Family planning, Antenatal care, Gestational diabetes mellitus

## Abstract

**Introduction:**

Family planning (FP) is important in reducing maternal morbidity and mortality as well as foetal and neonatal complications. This study aimed to determine the intention to practise FP among antenatal women at risk of gestational diabetes mellitus (GDM) in the Klang Health District and its associated factors.

**Methods:**

A cross-sectional study was conducted at four government health clinics in the Klang Health District. A total of 431 antenatal women at risk of GDM were recruited using systematic random sampling. A validated self-administered questionnaire was used to assess knowledge, attitude, previous practice and intention to use FP after delivery. Multiple logistic regression (MLR) was used to determine the factors associated with the intention to practise FP

**Results:**

Approximately 64.7% (n=279) of the respondents intended to practise FP MLR showed that the factors associated with the intention to practise FP were Malay ethnicity (odds ratio [OR]=3.319, 95% confidence interval [CI]=1.431–7.697), low income (OR=2.174, 95% CI=1.317–3.588), good knowledge (OR=2.591, 95% CI=L008–6.174) and good previous practice (OR=3.956, 95% CI=1.428-9.052).

**Conclusion:**

The prevalence of the intention to practise FP among antenatal women at risk of GDM was 64.7%. Malay antenatal women from low-income households with good knowledge and previous practice were more likely to have the intention to practise FP after delivery. Thus, interventions targeted at non-Malay women and measures to improve their knowledge might help improve the intention and uptake of FP among these women.

## Introduction

Gestational diabetes mellitus (GDM) is a condition wherein women develop glucose intolerance resulting in hyperglycaemia during pregnancy.^1^ The adverse outcomes of GDM involve both the mother and baby. The risks of macrosomia, childhood obesity and glucose intolerance are increased while women with GDM have a higher risk of developing type 2 diabetes mellitus (DM) and cardiovascular disease in the future.^2^

The prevalence of GDM varies among regions, with the lowest rate (7.1%) noted in North America and the Caribbean and the highest rate (27.6%) in Southeast Asia.^3^ In Malaysia, GDM is prevalent, with the rate ranging from 18.3% to 27.9%.^4^

Family planning (FP) allows couples to anticipate and attain their desired number of children. It helps women avoid unintended and closed-spacing pregnancies.^5^ Effective FP methods among reproductive-age women can reduce unintended pregnancies and neonatal mortality, hence leading to a reduction in maternal mortality and morbidity.^6,7^ Thus, the National FP Programme was launched in 1966 to improve maternal and child health outcomes. In the Fifth Malaysian Population and Family Survey conducted in 2014, the prevalence of FP in Malaysia was only 52.2% in married women aged 15-49 years, and only 34.3% used modern methods of FP.^8^ These rates were much lower than the global contraceptive prevalence rate (63%) and the rates reported in neighbouring countries such as Indonesia (61.4%), Thailand (81.1%), Singapore (62.0%) and Vietnam (79%).^8^

FP is an important measure used in preconception care, especially for women with high-risk pregnancies who have chronic diseases such as DM and hypertension. FP could allow these high-risk women to optimise their medical conditions before conception for better maternal and birth outcomes.^9^

High-risk pregnant women should be given adequate counselling about FP during prepregnancy and postpartum periods.^10^ A study conducted in a high-risk pregnancy clinic in Turkey from 2009 to 2011 showed that women who had experienced high-risk pregnancy were more aware of the importance of FP and ready to accept counselling regarding FP.^11^ However, mothers who have no medical illness, but are at risk of developing GDM, are often given less attention than women who have established chronic diseases such as hypertension and DM. The risk factors of GDM among women include a family history of DM, overweight, history of GDM and history of macrosomia.^12^

For women at risk of developing GDM and pregnancy complications, good FP is equally important as it is for mothers with chronic medical diseases. To date, previous studies have mainly focused on FP practice among women with established medical illnesses such as DM and hypertension. In these studies, the prevalence of the use of FP has been reported to be 20%-35% in women with hypertension and 5%-90% in women with DM.^13-15^ To the best of our knowledge, no study has evaluated mothers who are well but at risk of GDM. Therefore, this study aimed to assess the factors contributing to the intention to practise FP among antenatal women at risk of developing GDM in the Klang Health District. The findings are expected to provide insights into the factors that could be targeted to improve the intention and uptake of FP among these women.

## Methods

### Study design and population

This cross-sectional study was conducted from July to September 2022 at four government health clinics in the Klang Health District: Klinik Kesihatan Pelabuhan Klang, Klinik Kesihatan Kapar, Klinik Kesihatan Pandamaran and Klinik Kesihatan Anika. Malaysian antenatal women at risk of GDM and aged >18 years were included in the study. Conversely, antenatal women who were unable to understand Malay or English, had acute emergencies and had established type 2 DM were excluded from the study.

### Sample size calculation

This study used an adjusted sample size formula for the required population. Based on the official registry of the Modified Glucose Tolerance Test (MGTT) for antenatal mothers in the Klang District Health Office from October to December 2021, the number of antenatal mothers was 3790; conversely, the number of antenatal mothers at the selected health clinics was 2253.

We used the following formula to calculate our sample size: n=[z^2^*p*(1-p)/e^2^]/[1 + (z^2^*p*(1-p)/(e^2^*N))], where n is the sample size; z, z-score, associated with the level of confidence; p, sample proportion, expressed as a decimal; e, margin of error, expressed as a decimal; and N, population size.

z = 1.96 for a confidence level (α) of 95%

p = 0

e = 0.05


$$\text{n}=\frac{\left[1.96^{2}*0.5*\left(1-0.5\right)/0.05^{2}\right]}{\left[1+\left(1.96^{2}*0.5*\left(1-0.5\right)/\left(0.05^{2}*2253\right)\right)\right]}$$

The sample size calculated was 329. With a non-response rate of 30%, the estimated sample size required was 427.

### Data collection

Respondents were recruited via systematic random sampling. The sampling frame was the appointment list for the MGTT among antenatal women at risk of GDM. The interval of selection calculated was 5. The first respondent was the fifth patient who was selected using simple random sampling from the first five patients. Subsequent respondents were selected afterward in intervals of five from the first respondent (i.e. 5 th, 10th, 15th and so on). Selected respondents who refused to participate in the study were replaced by the next fifth respondents.

This study used a self-administered questionnaire in bilanguage (English and Malay) versions. The first part of the questionnaire assessed sociodemographic details, consisting of 14 questions about age, marital status, ethnicity, educational level, household income, weight, height, parity and antenatal history.

The second part of the questionnaire was a validated questionnaire adapted from the local literature that measured knowledge, attitude and previous practice of FP (Appendix 1).^16^ The reported internal consistency of the items was good based on Cronbach’s alpha values of 0.890, 0.708 and 0.802 for knowledge, attitude and previous practice, respectively.^16^ The knowledge section comprised 12 items covering the methods, benefits and side effects of FP Each item was measured using a 3-point Likert scale, with the response options being ‘yes’, ‘no’ and ‘do not know’. A score of 1 was given to every correct answer and 0 to every incorrect answer or ‘do not know’.^16^ For the attitude section, 15 items covering the attitudes towards age at the first child conception, satisfaction with the current contraceptive method, childbirth spacing, ideal number of children planned, discussion with husband, religious and cultural beliefs and husband’s objection in using contraception were assessed using a 5-point Likert scale. For positive statements, a score of 5 was given for ‘strongly agree’, 4 for ‘agree’, 3 for ‘neutral’, 1 for ‘disagree’ and 0 for ‘strongly disagree’. For negative statements, the scoring was reversed. For the practice section, seven items were used to assess previous practice of FP, scored using a 5-point Likert scale. A score of 5 was given for ‘always’, 4 for ‘usually’, 3 for ‘sometimes’, 2 for ‘seldom’ and 1 for ‘never’.^16^

The last part of the questionnaire was an independent question that asked about the intention to practise FP at any time after the delivery of the current pregnancy. A 5-point Likert scale was used to score the response: ‘very likely’, ‘likely’, ‘neutral’, ‘unlikely’ and ‘very unlikely’.

### Statistical analysis

Statistical analysis was performed using the IBM SPSS Statistics for Windows, version 28 (IBM Corp., Armonk, N.Y., USA). The dependent variable was the intention to practise FP after the current pregnancy. The independent variables were the sociodemographic background, knowledge level, attitude level and previous practice level.

The knowledge level was classified into three categories based on the total knowledge score obtained according to the recommendation from previous literature^16^: good (total knowledge score of 10–12), moderate (total knowledge score of 7–9) and poor (total knowledge score of <7). For the attitude section, the score for each item was summed. Respondents who scored less than 45 were categorised as having a poor attitude; 45–59, a moderate attitude; and ≥60, a good attitude.^16^ For the previous practice of FP, the total scores were interpreted as follows: good (total score of 26–35), moderate (total score of 21–25) and poor (total score of ≤20) practice. For the intention to practise FP after delivery, ‘very likely’ and ‘likely’ were grouped into the ‘yes’ category (have the intention to practise FP after delivery) and ‘natural’, ‘unlikely’ and ‘very unlikely’ into the ‘no’ category (does not have the intention to practise FP after delivery).

Bivariate analysis using the chi-square test was conducted to determine any significant association between the dependent and independent variables. Data were further analysed using simple and multiple logistic regression analyses to determine the predictors of the intention to practise FP after delivery. The significance level was set at P<0.05.

## Results

A total of 431 antenatal women at risk of GDM were approached, and all of them agreed to participate. Thus, the response rate was 100%.

### Intention to practise FP after the current pregnancy

Approximately 64.7% (n=279) of the respondents reported that they had the intention to practise FP after delivery. Table 1 illustrates the sociodemographic characteristics as well as the levels of knowledge, attitude and previous practice of FP of the respondents during their antenatal period. The mean age was 29.6 (SD=4.790) years. About 76.1% of the respondents were Malay. One-tenth (10.9%) of the respondents were grand multiparous, and 32% had poor previous pregnancy spacing. Of the respondents, 65.2% came from a low-income household (<RM 4850 per month). Nearly half (49.9%) of the respondents had a secondary educational background, similar to their husband’s counterparts (53.8%). For the risk factors of GDM, 43.2% had a body mass index indicating obesity at booking; 26% had a history of GDM; 42.9% had a family history of DM; 1.2% had a history of macrosomia; and 2.6% had other underlying medical conditions that necessitated the need for GDM screening.

**Table 1:** Sociodemographic characteristics and levels of knowledge, attitude and previous practice of family planning among all 431 respondents.

Variable	n	%	Mean
**Age**	-	-	29.6
**Body mass index at booking** <27.5 kg/m^2^ (non-obese)	245	56.8	-
≥27.5 kg/m^2^ (obese)	186	43.2	
**Parity**			
Non-grand multiparity	384	89.1	-
Grand multiparity	47	10.9	
**Ethnicity**			
Malay	328	76.1	
Chinese	34	7.9	-
Indian	47	10.9	
Others	22	5.1	
**Educational level**			
Primary	9	2.1	
Secondary	215	49.9	-
Tertiary	151	35.0	
Postgraduate	56	13.0	
**Partner’s educational level**			
No formal education	4	0.9	
Primary	17	3.9	
Secondary	232	53.8	-
Tertiary	139	32.3	
Postgraduate	39	9.0	
**Household income per month**			
<RM 4850 (B40)	281	65.2	
RM 4851-10,970	110	25.5	-
>RM 10,970	40	9.3	
**History of pregnancy within 2 years of the last childbirth (poor spacing)**			
Yes	138	32.0	-
No	293	68.0	
**History of gestational diabetes mellitus**			
Yes	112	26.0	-
No	319	74.0	
**First-degree relative with diabetes mellitus**			
Yes	185	42.9	-
No	246	57.1	
**History of macrosomia (childbirth weight of >4 kg)**			
Yes	5	1.2	-
No	426	98.8	
**Current obstetric problem**			
Chronic hypertension	3	0.7	
Pregnancy-induced hypertension	1	0.2	
Polyhydramnios	4	0.9	-
Use of steroids	3	0.7	
No other medical problem	420	97.4	
**Knowledge level**			
Good (score of 10-12)	42	9.7	
Moderate (score of 7-9)	158	36.7	-
Poor (score of <7)	231	53.6	
**Attitude level**			
Good (score of ≥60)	133	30.9	
Moderate (score of 45-59)	284	65.9	-
Poor (score of <45)	14	3.2	
**Previous practice level**			
Good (score of >25)	49	11.4	
Moderate (21-25)	77	17.9	-
Poor (score of ≤20)	305	70.8	

Regarding knowledge of FP, only 9.7% of the respondents had a good knowledge level; 36.7%, moderate knowledge level; and 53.6%, poor knowledge level. For attitude towards FP, 30.9% had good attitude; 65.9%, moderate attitude; and only 3.2%, poor attitude. The majority of the respondents had poor previous practice of FP, whereas 70.8% did not previously practise FP

### Relationship of the intention to practise FP with the sociodemographic characteristics and levels of knowledge, attitude and previous practice of FP

The association between the respondents’ sociodemographic backgrounds as well as levels of knowledge, attitude and previous practice and the intention to practise FP after delivery of their current pregnancy was examined using the chi-square test (Table 2). The significant factors identified were parity (P=0.011), ethnicity (P<0.001), household income (P<0.001), knowledge level (P<0.001), attitude level (P=0.023) and previous practice level (P<0.001). Approximately 76.6% of the grand multiparous women and 57.3% of the non-grand multiparous women had the intention to practise FP. Among all ethnicities in Malaysia, 54.5% of the Malays, 34% of the Indians and 29.4% of the Chinese had the intention to practise FP. Household income had a significant association with the intention to practise FP. About 65.8% of the women from low-income households, 45.5% from middle-income households and 52.5% from high-income households intended to practise FP. Among the respondents having the intention to practise FP after delivery, the proportion of women with a good level of knowledge, attitude and previous practice of FP was significantly larger than that of women with moderate and poor levels of knowledge, attitude and previous practice of FP (Table 2).

**Table 2:** Relationship of the sociodemographic characteristics and levels of knowledge, attitude and previous practice of family planning with the intention to practise family planning after delivery.

Variable	With intention n (%)	Without intention n (%)	X^2^	P
Age	256 (59.4)	175 (40.6)	136 (35.6)	0.763^b^
**Body mass index**				
<27.5 kg/m2 (non-obese) ≥27.5 kg/m2 (obese)	141 (57.6) 115 (61.8)	104 (42.4) 71 (38.2)	0.626	0.371^a^
**Parity**				
Non-grand multiparity Grand multiparity	220 (57.3) 36 (76.6)	164 (42.7) 11 (23.4)	6.470	0.011^a^
**Ethnicity** Malay Chinese Indian Others	218 (54.5) 10 (29.4) 16 (34.0) 12 (54.5)	110 (33.5) 24 (70.6) 31 (66.0) 10 (45.4)	32.210	**<0.001^a^**
**Educational level** Primary Secondary Tertiary Postgraduate	6 (66.7) 124 (57.7) 98 (64.9) 28 (50.0)	3 (33.3) 91 (42.3) 53 (35.1) 28 (50.0)	4.409	0.221^a^
**Partner’s educational level** No formal education Primary Secondary Tertiary Postgraduate	3 (75.0) 11 (64.7) 138 (59.5) 81 (58.3) 23 (59.0)	1 (25.0) 6 (35.3) 94 (40.5) 58 (41.7) 16 (41.0)	0.679	0.954^a^
**Household income per month**				
<RM 4850 RM 4851-10,970 >RM 10,970	185 (65.8) 50 (45.5) 21 (52.5)	96 (34.2) 60 (54.5) 19 (47.5)	14.487	<0.001^a^
**Knowledge level** Good Moderate Poor	33 (78.6) 105 (66.5) 118 (51.1)	9 (21.4) 53 (33.5) 113 (48.9)		**<0.001^a^**
**Attitude level**				
Good	91 (68.4)	42 (31.6)		**0.023^a^**
Moderate	159 (56.0)	125 (44.0)		
Poor	6 (42.9)	8 (57.1)		
**Previous practice level**				
Good	43 (87.8)	6 (12.2)		**<0.001^a^**
Moderate	47 (61.0)	30 (39.0)		
Poor	166 (54.4)	139 (45.6)		

a Pearson chi-square test

b independent sample t-test

### Predictors of the intention to practise FP

Seven variables with P-values of <0.25 (ethnicity, educational level, household income, parity, knowledge level, attitude level and previous practice level) were included in the multiple logistic analysis. The analysis showed that four factors (i.e. ethnicity, household income, knowledge level and previous practice level) significantly increased the odds of having the intention to practise FP

The Malays had 3.319 times higher odds (odds ratio [OR]=3.319, 95% confidence interval [CI] = 1.431–7.697) of having the intention to practise FP after their current pregnancy than the Chinese. The respondents from low-income households had 2.174 times higher odds (OR=2.174, 95% CI=1.317–3.588) of having the intention to practise FP than those from middle-income households. Conversely, the respondents with good knowledge (OR=2.591, 95% CI= 1.008–6.174) and previous practice levels (OR=3.956, 95% CI=1.428–9.052) had 2.591 and 3.596 times higher odds of having the intention to practise FP, respectively, than those with poor knowledge and previous practice levels (Table 3).

**Table 3:** Predictors of the intention to practise family planning among antenatal women at risk of gestational diabetes mellitus.

	Simple logistic regression Crude OR (95% CI)	P	Multiple logistic regression Adjusted OR (95% CI)	P
Age	1.005 (0.965-1.046)	0.809	-	
**Body mass index**				
Obese	0.837 (0.567-1.236)	0.371		
**Ethnicity** Chinese Malay Indian Others	1.00 4.756 (2.197-10.298) 1.239 (0.478-3.213) 2.880 (0.942-8.803)	**<0.001^#^** 0.660 0.064	1.00 3.319 (1.431-7.697) 1.189 (0.419-3.375) 2.589 (0.772-8.680)	0.005^*^ 0.774 0.123
**Educational level** Postgraduate Primary Secondary Tertiary	1.00 2.0 (4.555-8.800) 1.363 (0.756-2.457) 1.849 (0.993-3.442)	0.359 0.304 **0.053^#^**	1.00 2.926 (0.617-13.869) 1.207 (0.623-2.336) 1.349 (0.683-2.665)	0.176 0.577 0.388
**Partner’s educational level** Tertiary No formal education Primary Secondary Postgraduate	1.00 2.148 (0.218-21.173) 1.313 (0.459-3.753) 1.051 (0.686-1.611) 1.029 (0.500-2.118)	0.513 0.612 0.819 0.937	-	
**Household income status** Middle Low High	1.00 2.312 (1.476-3.623) 1.326 (0.642-2.739)	**<0.001^#^** 0.445	1.00 2.174 (1.317-3.588) 1.234 (0.564-2.697)	0.002^*^ 0.599
**Parity**				
Grand multiparity	2.440 (1.206-4.937)	**0.130^#^**	2.179 (1.018-4.663)	0.45
**Knowledge level** Poor Moderate Good	1.00 1.897 (1.248-2.885) 3.511 (1.608-7.667)	**0.003^#^** **0.002^#^**	1.00 1.474 (0.926-2.349) 2.591 (1.008-6.174)	0.102 0.032^*^
**Attitude level** Poor Moderate Good	1.00 1.696 (0.574-5.015) 2.889 (0.943-8.853)	0.340 0.063	1.00 1.828 (0.590-5.667) 2.242 (0.682-7.370)	0.296 0.184
**Previous practice level** Poor Moderate Good	1.00 1.312 (0.787-2.186) 6.001 (2.481-14.517)	0.297 **<0.001^#^**	1.00 1.164 (0.667-2.030) 3.596 (1.428-9.052)	0.593 0.007^*^

CI, confidence interval; OR, odds ratio

# P<0.25

* P<0.05

*The model fitness was assessed using the Hosmer–Lemeshow test; the value was 0.966, indicating that the model fitted well. The model assumption was met. There was neither significant interaction nor multicollinearity (IVF ranging from 1 to 10) found among the independent variables. The model explained 23.1% (Nagelkerke R^2^) of the variance in the factors associated with the intention to practise FP after delivery. The classification table demonstrated that the model could correctly classify 68.4% of the respondents; 80.5% were correctly predicted to have the intention to practise FP and 50.9% not to have the intention to practise FP. The models correctly identified 73.8% of the respondents who had the intention to practise FP (AUC=, 0.727% CI=0.679–0.774P<0.024), and there were no influential outliers noted*

## Discussion

This study found that the prevalence of the intention to practise FP among the antenatal women at risk of GDM was 64.7%. The prevalence is higher in this study than in studies conducted in Serdang and a suburban area in Terengganu (38.4% and 38.7%, respectively).^17,18^ This finding may be attributed to the differences in the characteristics of the study population. The present study included high-risk antenatal mothers, whereas other studies involved the general population.

Herein, the antenatal women from lower-income households had 2.174 times higher odds of having the intention to practise FP than those from middle-income households. This finding is supported by that of Rohana and Khalili that when the opportunity cost of raising children increases, households prefer having fewer children.^19^ However, a different study reported inconsistent findings regarding the association between financial status and FP practice. Other local and international studies revealed that income significantly influenced FP practices and suggested that women with higher incomes may have better access to FP services.^11,16^ Conversely, a study conducted in Serdang found that income was not a significant factor of FP practices.^18^

In regard to ethnicity, the Malays were found to have higher odds of having the intention to practise FP after delivery than the Chinese.

A study conducted in 2022 showed a significantly higher percentage of the intention of Muslims to practise FP, where most Malays were Muslim. This fact can be translated as the likelihood that Malays have the intention to practise FP.^20^ This may be the result of the official legislation by bodies of fatwa in Malaysia together with the strenuous effort of the health ministry in promoting the benefits of FP among Muslim couples.

This study also demonstrated that good knowledge of FP had a significant association with the intention to practise regardless of educational background. The antenatal women with good knowledge were 2.591 times more likely to have the intention to practise FP than those with poor knowledge. Women who have good knowledge and are experienced in FP might be more concerned about the quality instead of the quantity of their children and thus have a greater intention to practise FP.^15,21^ Our study also showed that among the women who had no intention to practise FP after delivery, the proportion of women who had poor knowledge (48.9%) was larger than that of women who had moderate (33.5%) and good knowledge (21.4%). Thus, it is important to educate antenatal mothers regarding FP particularly regarding its mode of action, side effects, cost and overall benefits, since better knowledge about FP can positively impact their intention and uptake of FP.

We found that a good level of previous FP practice was a significant factor associated with the intention to practise FP. This result is in line with a report in Ghana and an online survey finding on FP utilisation among adults in Malaysia showing that previous experience of using FP significantly affected postpartum use of FP.^21,22^ This could be attributed to the fact that women who previously used FP methods might have more knowledge and understanding and thus were more likely to adopt such methods after delivery than those who did not.

Regarding attitudes towards FP, a local study showed significant positive attitudes towards FP.^18^ However, our study found that the attitude level of the antenatal women was not a predictor of the intention to practise FP Similarly, a study performed in Pakistan reported that 85% of women had positive attitudes towards FP, but only 47% of them practised FP.^23^ In another study conducted in Northern Turkey, the prevalence of using FP was relatively high (85.8%) among women despite having negative attitudes.^24^ Other previous studies also reported that attitude might not determine a person’s behaviour, as there might be different perceptions and responses from individuals.^25,26^ In addition, other factors or circumstances such as personal characteristics and strength may influence the intention to practise FP regardless of the attitude towards FP^25^

### Strength and limitation

The study included respondents from various sociodemographic backgrounds, reflecting diversity among the Malaysian population.^27^ However, this study did not measure the exact uptake of FP among these high-risk women after their delivery due to constraints of resources. The intention to practise FP might not translate to real action. Further cohort studies are needed to verify the relationship between the intention and uptake of FP.

## Conclusion

The prevalence of the intention to practise FP among women at risk of GDM was 64.7%. The factors found to be significantly associated with the intention to practise FP were ethnicity, income status, knowledge level and previous practice level. Interventions addressing these factors might help improve the intention and uptake of FP among these women.

## Data sharing statement

Data are available upon request from the corresponding author.

**How does this paper make a difference in general practice?**

- This study provides insights into the intention of antenatal women at risk of gestational diabetes mellitus (GDM) to practise family planning (FP) after delivery.
- The prevalence of the intention to practise FP among antenatal women at risk of GDM was moderate (64.7%). Malay antenatal women from low-income households with good knowledge and previous practice were more likely to have the intention to practise FP after delivery.
- Interventions targeting non-Malay women and measures aiming to enhance their knowledge might help improve the intention and uptake of FP among high-risk women.
